# Vincristine alleviates adriamycin-induced nephropathy through stabilizing actin cytoskeleton

**DOI:** 10.1186/s13578-016-0129-z

**Published:** 2017-01-03

**Authors:** Lei Yin, Youying Mao, Hejie Song, Ye Wang, Wei Zhou, Zhen Zhang

**Affiliations:** 1 Department of Nephrology and Rheumatology, Shanghai Children’s Medical Center, School of Medicine, Shanghai Jiaotong University, Shanghai, China; 2 Institute of Pediatric Translational Medicine, Shanghai Children’s Medical Center, School of Medicine, Shanghai Jiaotong University, Shanghai, China; 3 Pediatric Congenital Heart Disease Institute, Shanghai Children’s Medical Center, School of Medicine, Shanghai Jiaotong University, Shanghai, China

**Keywords:** Nephrotic syndrome, Vincristine, Actin cytoskeleton, Focal adhesion kinase, Integrin

## Abstract

Antimicrotubule agent vincristine (VCR) has long been known as an alternative treatment for frequent relapse nephrotic syndrome and steroid-dependent nephrotic syndrome (SDNS). However, the mechanism is unknown. Here we found that VCR at a dosage much lower than that as an antimicrotubule agent can alleviate adriamycin (ADR)-induced proteinuria and podocyte foot process effacement. In cultured podocytes, VCR prevents ADR-induced actin fiber disorganization. In both in vitro and in vivo models, VCR suppresses ADR-induced overexpression of α3β1 integrin and focal adhesion kinase (FAK). These data suggest that VCR may relieve ADR-induced nephropathy through inhibiting injury-induced activation of integrin outside-in signaling to prevent actin cytoskeleton remodeling. Hence, our work reveals a novel role of VCR in regulating actin fiber assembly and provides first evidence on the therapeutic mechanism of VCR on nephrotic syndrome.

## Background

Minimal change nephrotic syndrome (MCNS) is a common renal disease in childhood. Although about 90% of children with MCNS respond to prednisone treatment, nearly 30% of them have frequent relapses or develop steroid-dependent nephrotic syndrome. Steroid toxicity following repeated steroid treatment demands alternative treatments in those patients [1]. However, current corticosteroid-sparing agents prescribed for children with frequent relapse nephrotic syndrome and SDNS have their own shortcomings, such as high frequent relapses after levamisol or mycophenolic acid discontinuing, risk for infertility after repeated courses of alkylating agents and nephrotoxicity of calcinurin inhibitors [1]. Therefore, it is necessary to search for alternative treatments for patients with frequent relapse nephrotic syndrome and SDNS.

Vincristine (VCR) is a vinca alkaloid that has played an important role in chemotherapy of malignant diseases for 3 decades. The beneficial effects of VCR in nephrotic syndrome associated with hematological malignancy have been noted for many years [2]. But these therapies were consisted of a combination of VCR, sterioids and other cytotoxic agents and it was unclear whether the remission of nephrotic syndrome was directly related to VCR. Goonasekera et al. reported two cases of primary focal segmental glomerulosclerosis (FSGS) that responded to VCR alone [2]. Given those reports, we once treated 12 SDNS patients with intravenous VCR, all of whom had previously completed at least one 8-week course of cyclophosphamide but still had frequent relapses. Results showed that VCR treatment in a subset of children with challenging SDNS could not only relieve proteinuria but also reduce relapse frequency. Moreover, side effects were minimal in most cases [3]. It indicates that VCR may be a valuable alternative treatment for idiopathic nephrotic syndrome patients. However, it’s not clear how VCR relieves symptoms of nephrotic syndrome.

Here we demonstrated that VCR could decrease proteinuria in ADR nephropathy rats after 2-week treatment and relieve hypoalbuminemia and hypercholesterolemia after 4-week treatment. Consistently, VCR rescued podocyte foot process effacement induced by ADR. VCR treatment could stabilize actin cytoskeleton structure and rescue the morphology of ADR-injured podocytes. Molecular analyses indicate that the therapeutic effect of VCR may be mediated by inhibiting the pathological overexpression of α3β1 integrin and FAK in ADR-induced nephropathy.

## Methods

### ADR nephropathy rat model and treatment with VCR

ADR nephropathy was induced in male SD rats weighing 200 ± 20 g by tail vein injection with single dose of ADR (7.5 mg/kg body weight, dissolved in 0.9% NaCl) [4]. As normal control group, rats were injected with equal volume of 0.9% NaCl. Four weeks later, ADR nephropathy rats were divided in two groups. One group was injected with VCR 0.2 mg/kg body weight twice per week for 4 weeks. The other group was injected with equal volume of 0.9% NaCl. Urine was collected from each rat in metabolic cage to determine 24 h protein level. It was done weekly after ADR injection for the first two weeks and then once for every two weeks. Blood samples were obtained in chloral hydrate anaesthetized rats at 2, 4 and 8 weeks after ADR injection. Plasma was extracted for serum albumin and cholesterol measurement.

### Podocyte cell culture

Immortalized mouse podocyte cell line was kindly provided by Professor Jianghua Chen (The First Affiliated Hospital of Zhejiang University, School of Medicine). The immortalized podocytes were cultured for 5 days at 33 °C in 5% CO_2_ under permissive condition [RPMI 1640 (Sigma) with 10% fetal bovine serum (Gibco), 10U/ml of recombinant mouse γ-interferon (Peprotech) and 100U/ml penicillin/streptomycin (Gibco) on type I collagen (Sigma)-coated BD dishes]. They were subsequently cultured at 37 °C in 5% CO_2_ under restrictive condition (permissive condition without γ-IFN) to induce differentiation. It took 12–14 days to induce differentiation. After podocytes were well-differentiated, they were incubated with 0.5 µM ADR to induce cell injury. For VCR treatment group, 5 nM VCR was added 1 h after ADR injury. Cells were further cultured for 24 h before the following processes.

### F-actin immunofluoresence staining

100 nM working stock of Actin-stain™ 488 phalloidin was prepared by diluting 3.5 μl of 14 μM labeled stock phalloidin (Cytoskeleton) into 500 μl of PBS. Cells were gently washed by PBS after removing culture media at room temperature (RT). After 3.7% paraformaldehyde (PFA) fixation, cells were rinsed once with PBS and permeabilized by 0.5% Triton X-100/PBS. Cells were rinsed once with PBS and then 100 nM Actin-stain™ 488 phalloidin was incubated with cells at RT in dark for 30 min. After washing three times in PBS, cells were counter-stained by DAPI. Images were obtained with DM6000 upright microscope (Leica). Fifty high magnification fields from each sample were randomly chosen for the measurement of cell area and actin fiber number. All the analyses were done with Image-Pro Plus. Three separate experiments for each group were analyzed.

### Quantitative PCR

Total RNA was extracted from the podocytes and kidney cortex with Trizol (Invitrogen). 1ug total RNA from each sample was reverse transcribed with PrimeScript RT Kit (TaKaRa) to synthesize cDNA. Next, 1ul cDNA was used for PCR amplification with SYBR Green PCR Master Mix (TaKaRa). Reactions were performed on a 7900HT Fast Realtime PCR system (ABI). Gapdh was used as internal normalizer for all the examined genes. At least three samples from each group were used for qPCR analysis. Primers were designed using the Primer Express software (Primer Premier 5.0) based on the Gene Bank accession numbers. Primer sequences are:

Gapdh: 5′-CTCATGACCACAGTCCATGC; 5′-CACAATTGGGGGTAGGAACAC; Podocin: 5′-ACCTTTCCATGAGGTGGTAAC; 5′-CTGGATGGCTTTGGACAC Nephrin: 5′-CTTGTTGTCCGATTTGCCCC; 5′-CCTGGGCTGCAGACACATTA; Synaptopodin: 5′-GCTGGAGCTTTGGGCCG; 5′-GTTGAAGAGCTGGACCCCTC; Podocalyxin: 5′-TACATCCAAACCGACAGGCA; 5′-GGCTGTAGTGGTGTGGAGAC; FAK: 5′-ATACACCATGCCCTCAACCAG; 5′-GGTCAAACTGGCGCATTGTT α3-integrin: 5′-ATCCACAGCAATGGGTCCTG; 5′-GGAACAGGTCAACACGGTCT; β1-integrin: 5′-TGTCCTACTGGTCCCGACAT; 5′-TTTTCACCCGTGTCCCACTT

### Western blot

Podocytes and kidney cortex were lysed with RIPA Lysis Buffer (Beyotime) supplemented with protease inhibitor (Sigma) and phosphatase inhibitor (Roche). After determination of protein concentration with BCA protein assay kit (Bio-Rad), 20 µg protein per sample were denatured by boiling at 96 °C for 5 min in sample buffer. Equivalent amounts of protein were loaded and electrophoresed in SDS-PAGE gels. After electrophoresis, gels were transferred to nitrocellulose (NC) filter membranes. Next, membranes were blocked with mouse Anti-FAK (BD Transduction) or anti-phospho-FAK (Tyr397, Millipore) for 2 h. After washing with PBS, membranes were incubated with IRDye 800-conjugated goat anti-rabbit IgG secondary antibody (Rockland) for half an hour. Immunoreactive bands were visualized with a Licor Odyssey Infrared Imaging System (LI-COR Biosciences).

### Histology

Rats were anaesthetized by chloral hydrate and were sacrificed by cervical vertebrae. Kidneys were removed from the bodies. The kidney cortex were isolated and fixed in 10% neutral buffered formalin for hematoxylin and eosin staining or fixed in 2.5% glutaraldehyde for electron microscopy.

### Statistical analysis

Results were analyzed for statistically significant differences using SPSS 16.0 software. Data were presented as mean ± standard deviation (SD) and tested by one-way ANOVA. *p* < 0.05 were considered significantly.

## Results

### VCR ameliorates symptoms of ADR-induced nephropathy and rescues foot process effacement

**Fig. 1:**
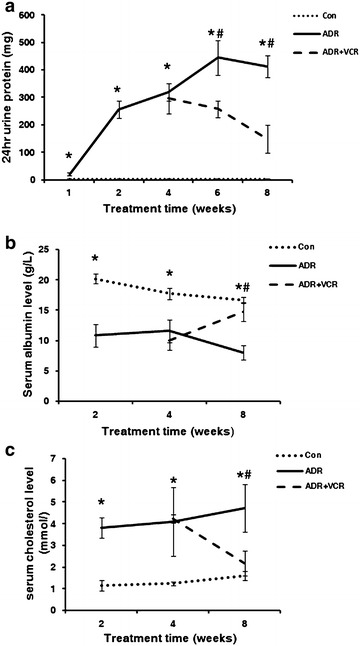
VCR mitigates ADR-induced nephropathy. **a** Line chart of 24 h urine protein. **b** Line chart of serum albumin level. **c** Line chart of serum cholestoral level. Data are presented as Mean ± SD. *p < 0.05, comparison within each group. ^#^p < 0.05, comparison between ADR and ADR + VCR groups

**Fig. 2:**
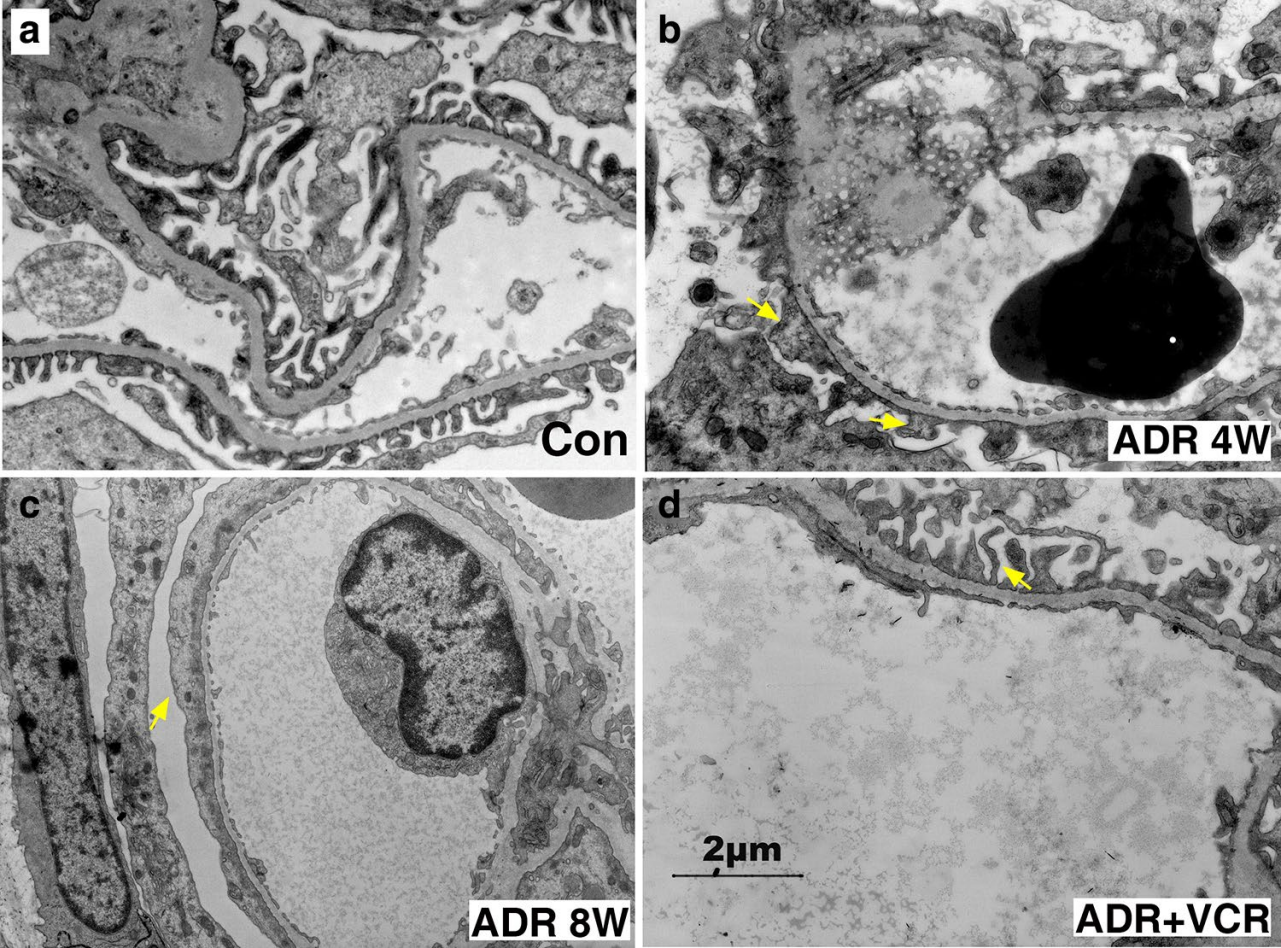
VCR rescues ADR-induced foot process effacement of podocytes. **a**–**d** EM images of podocytes. **a** Slit-like foot processes (*arrows*) in control group. **b** The majority of foot processes are fused with each other (*arrows*) after 4-week ADR treatment. **c** Fused foot processes of podocytes form a flat sheet (*arrow*) to wrap around capillary vessel after 8-week ADR treatment. **d** Slit-like foot processes (*arrow*) reappear after 4-week VCR treatment

**Fig. 3:**
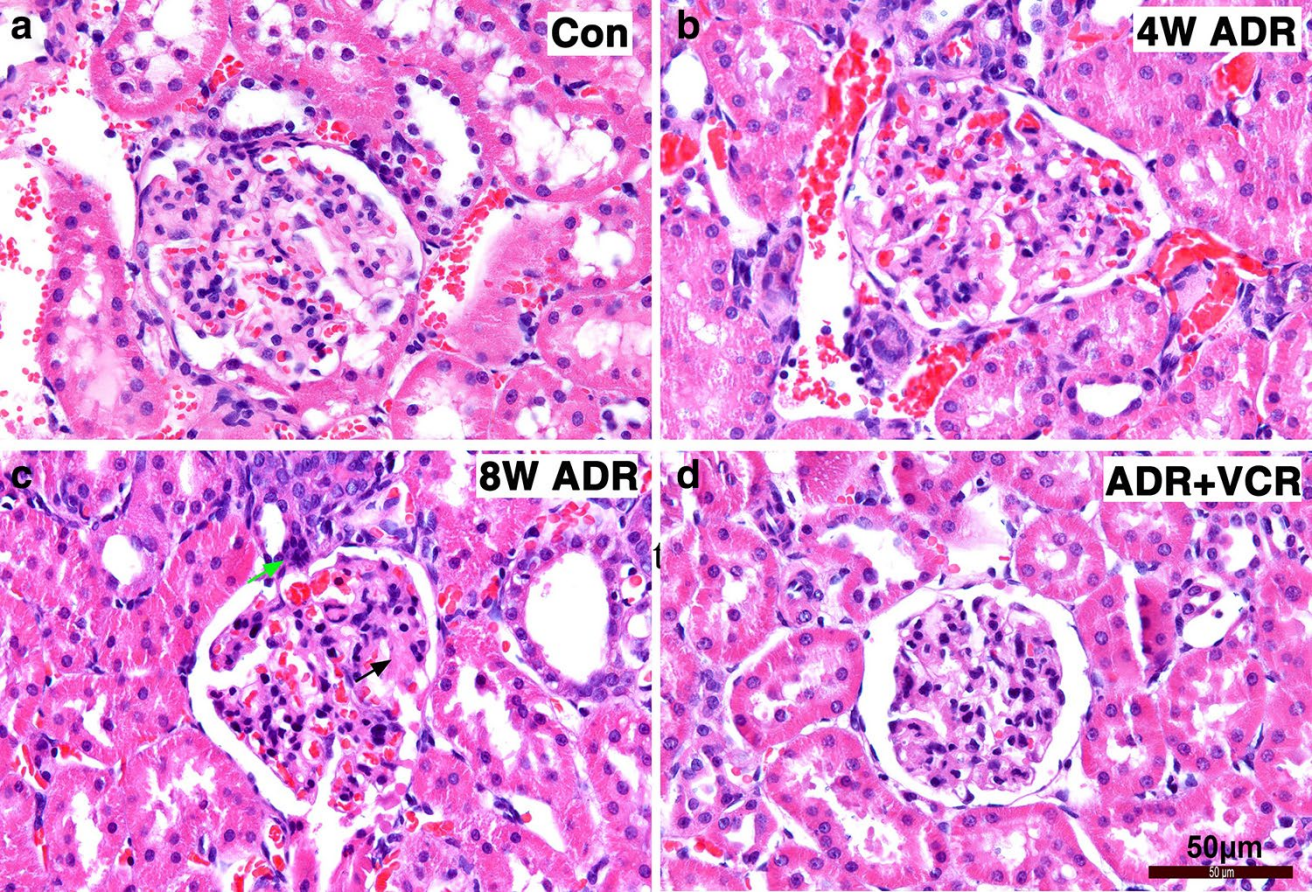
Histological change of glomeruli. **a**–**d** HE staining of kidney section. **a** Control. **b** Four-week ADR treatment group. **c** A slight mesangial matrix deposition (*black arrow*) and interstitial cell infiltration (*green arrow*) in glomeruli at 8-week ADR treatment group. **d** ADR and VCR treatment group. *Scale bar* 50 µm

As previously shown [3], VCR has been used to treat SDNS patients. To understand how VCR works, we first tested whether VCR could treat ADR-induced nephropathy in rat. According to the established protocol [4], we treated SD rats with 7.5 mg/kg body weight ADR to induce nephropathy. One week after injection, treated rats had already shown significant urine protein (Fig. 1a). The level of 24 h urine protein rapidly increased at the second week (Fig. 1a). Its level reached peak at the sixth week and was maintained at the high level from then on (Fig. 1a). On the other hand, serum albumin level significantly decreased while serum cholesterol level significantly increased at all the time points after ADR injection (Fig. 1b, c). Furthermore, we examined the ultrastructural change of kidney cortex under electron microscope. Effacement of glomerular podocyte foot processes was obvious at 4 weeks after ADR injection (arrows in Fig. 2b) and became more extensive 4 weeks later (arrow in Fig. 2c). HE staining of renal sections at 4 weeks after ADR treatment didn’t reveal any abnormal change (Fig. 2b). However, HE staining of renal sections at 8 weeks after ADR treatment showed that glomeruli developed very mild histological changes such as mesangial matrix expansion (black arrow in Fig. 3c) and cell infiltration in interstitial area (green arrow in Fig. 3c). These data indicate successful establishment of MCNS model.

Starting from 4 weeks after ADR injection, rats in VCR treatment group were administrated with 0.2 mg/kg body weight VCR, which was converted from clinical dosage 1 mg/m^2^ according to dose conversion between human and rat. At the beginning of VCR treatment, there was no difference in 24 h urine protein level between ADR and VCR group (Fig. 1a). However, 24 h urine protein level decreased significantly after two-week VCR treatment and continued to decrease afterwards (Fig. 1a). Consistently, both serum albumin and cholesterol levels were recovered to normal ranges after 4 weeks’ treatment (Fig. 1b, c). Ultrastructurally, the majority of podocyte foot processes reappeared after 4-week VCR treatment (Fig. 2d). On cellular level, mesangial matrix deposition and cell infiltration in VCR treatment group were also attenuated (Fig. 3d). These data indicates that VCR can rescue effacement of podocyte foot process to ameliorate ADR-induced nephropathy.

### Vincristine stabilizes actin cytoskeleton to maintain podocyte morphology in ADR-injured podocytes

**Fig. 4:**
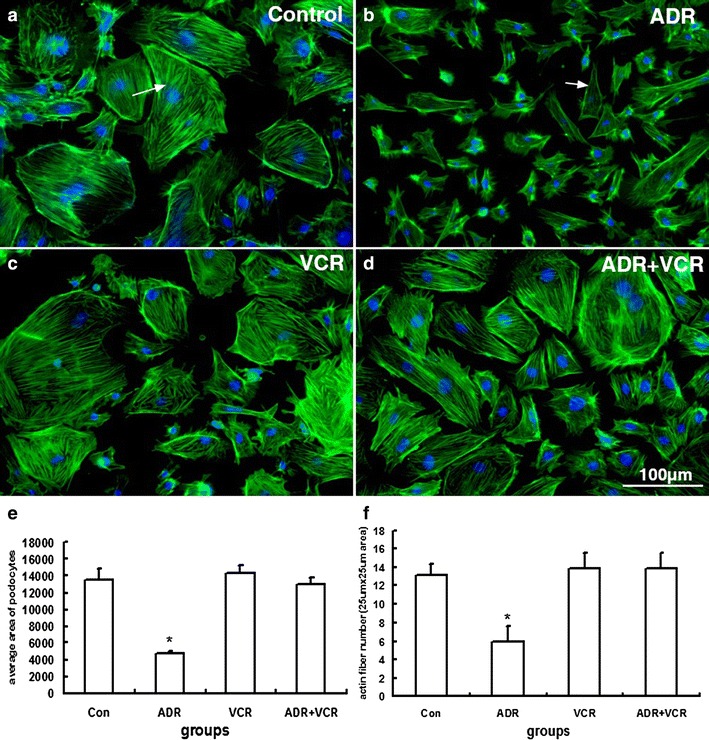
VCR alleviates ADR-induced disorganization of actin cytoskeleton. **a**–**d** Fluorescence microscope images of podocytes stained with Alexa 488 conjugated Phalloidin. **a** Well-organized linear actin fibers in control podocyte. **b** ADR treatment reduces the cell size of podocyte and diminished cytoplasmic actin fibers (*arrow*). **c** VCR treatment alone doesn’t change actin cytoskeleton distribution. **d** ADR-induced cellular changes are rescued by VCR treatment. **e** Quantitative comparison of cell size among different groups. **f** Quantitative comparison of actin fiber number among different groups. Data are presented as Mean ± SD. *p < 0.05

Podocytes are highly differentiated kidney cells playing an important role in maintaining the glomerular filtration barrier. Renal disorders that present with proteinuria are usually associated with marked foot process effacement of podocytes. Intact actin cytoskeleton is essential to maintain the normal physiology and unique morphology of podocyte. ADR directly induces actin fiber depolymerization of podocyte and thereby disrupt its normal function. To understand the therapeutic mechanism of VCR, we first examined the effect of VCR on the actin cytoskeleton organization. Normally, well-differentiated podocytes have an enlarged polygonal shape with non-polarized phenotype in culture and a dense meshwork of linear actin fibers span the entire cell (Fig. 4a). As reported before [5], treatment with 0.5 μM ADR for 24 h disrupted the normal morphology of podocytes. Cell shrank to spindle-like shape and cytoplasmic actin fibers were diminished (Fig. 4b).

VCR functions as an antimicrotubule agent and blocks cell mitosis at high concentration (≥40 nM) [6]. Since the dosage used for nephrotic treatment is much lower than that for cancer treatment, we first tested the effect of VCR on cultured podocytes at lower concentrations (1, 2, 5, 10 and 20 nM). We found that treatment with 1, 2 or 5 nM VCR for 24 h didn’t have any adverse effect on podocyte actin fiber and microtubule cytoskeleton (Fig. 4c and data not shown). Hence, we applied 5 nM VCR to test VCR effect on ADR injured podocyts. Indeed, 5 nM VCR treatment 1 h after ADR injury could stabilize actin fibers and maintain actin cytoskeleton distribution, thereby preventing the shrinkage of podocytes, (Fig. 4d). Quantitative measurements indicated that both the average area and actin fiber number of podocyte decreased significantly (*p* < 0.05) in ADR injured podocytes compared with the untreated podocytes, while there was no significant difference (*p* > 0.05) between the untreated and ADR + VCR groups (Fig. 4e, f). These results demonstrate that a low concentration level of VCR can rescue ADR-induced podocyte defect through stabilizing actin fiber formation.

### VCR reduces pathological overexpression of α3β1 integrin and FAK in ADR-induced nephropathy

**Fig. 5:**
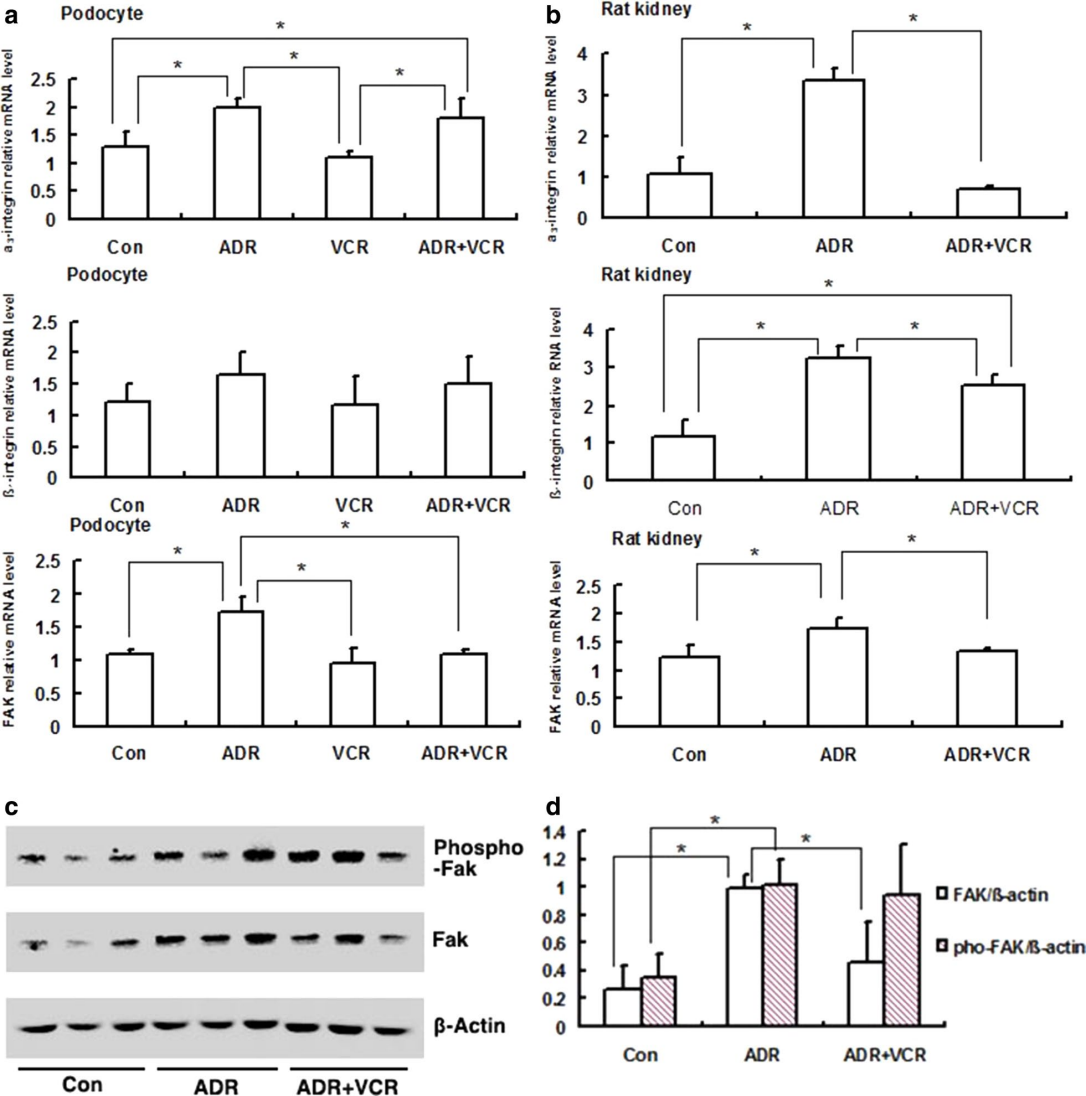
VCR represses ADR-induced overexpression of α3β1 integrin and FAK. **a** qPCR analysis of α3β1 integrin and FAK expression in podocytes. **b** qPCR analysis of α3β1 integrin and FAK expression in kidneys. **c** Western blot analysis of FAK and phospho-FAK in kidneys. **d** Quantification of FAK and phospho-FAK levels. Data are presented as Mean ± SD. *p < 0.05

To further investigate how VCR alleviates actin cytoskeleton disorganization in ADR-injured podocyte, we examined the expression of glomerular filtration barrier key components. Integrin-mediated outside-in signaling initiated by impairment of podocyte-GBM connections is one of the mechanisms causing foot process effacement and actin cytoskeleton disorganization [7]. α3β1 integrin and FAK are critical in this route [7, 8]. Quantitative PCR showed that α3β1 integrin expression levels in ADR nephropathy rats were significantly higher than those in control rats (*p* < 0.05), while VCR treatment significantly reduced α3β1 integrin overexpression (*p* < 0.05) (Fig. 5b). Similarly, we noted that FAK mRNA level in both cultured podocytes and rat kidney cortex samples was significantly increased after ADR injury (*p* < 0.05) and VCR treatment could significantly reduce FAK expression level (*p* < 0.05) (Fig. 5a, b). Consistently, we found that FAK protein level was also significantly increased in ADR-induced nephropathic rat kidneys (*p* < 0.05) and VCR treatment could rescue the pathological overexpression of FAK (*p* < 0.05) (Fig. 5c, d). The key event in FAK activation is the phosphorylation of Tyr397, which is required for Src binding and necessary for actin cytoskeleton remodeling [7]. However, we didn’t find the elevated FAK phosphorylation level was significantly rescued by VCR treatment (Fig. 5d). In addition, VCR treatment didn’t rescue ADR-induced expression changes for other critical regulators of actin dynamics, such as podocin, nephrin, synaptopodin and podocalyxin, both in podocyte and rat kidney cortex (data not shown). These data suggest that the therapeutical effect of VCR on actin fiber organization may be mediated through reducing pathological overexpression of α3β1 integrin and FAK.

## Discussion

VCR is a well-known antimicrotubule agent that induces tubulin self-association into coiled spiral aggregates to prevent microtubule assembly and therefore block cell mitosis [6]. Hence, it is widely used to treat human cancers [6]. Its beneficial effect on nephrotic syndrome of leukemia patients prompted nephrologists to use VCR as an alternative treatment for nephrotic syndrome [3]. But how VCR relieves nephrotic syndrome is unclear.

Nephrotic disorders with proteinuria are usually caused by marked foot process effacement of podocytes due to actin cytoskeletal disruption [7]. Indeed, we find that VCR can stabilize actin cytoskeleton in ADR-injured podocyte at a low dosage that doesn’t disrupt microtubules. It indicates that VCR has additional effect on cytoskeletons other than microtubule disruption. Interestingly, studies on vincristine resistance in malignant tumors show that expression changes in genes encoding actin cytoskeleton components are associated with the intrinsic and acquired VCR resistance [9], suggesting a complicated interaction between VCR and actin cytoskeleton. Here, we provide the first evidence indicating that a low concentration of VCR can stabilize actin fibers, although it is unclear whether VCR can directly bind actin fibers or not.

Actin cytoskeleton connects to extracellular matrix through focal adhesion in foot processes, which constitutes an integral component of the glomerular filtration barrier [7]. The maintenance and remodeling of these structures are regulated by integrin outside-in signaling modulated by FAK [8]. Podocyte FAK deletion or inhibition of FAK activation protect against proteinuria and foot process effacement induced by glomerular injury [8]. Our data suggest that the actin fiber-stabilizing effect of VCR may be mediated by suppressing overexpression of α3β1 integrin and FAK in both in vitro and in vivo models. Interestingly, we didn’t note that the level of activated form FAK Y397 was significantly reduced after VCR treatment. It indicates that the reduced FAK expression level, independent of FAK Y397 activation, is sufficient to alleviate proteinuria and foot process effacement. The segregation of FAK overexpression and FAK Y397 phosphorylation is also noted in the activation of cancer cell migration [10]. Our data provide novel insight into the complexity of FAK activation and downstream effect during the pathogenesis of podocyte injury, which requires further investigation. Further clarification of the therapeutic mechanism of VCR would help attract more attention to use it as a cheap and effective alternative treatment for nephrotic syndrome.

## Abbreviations

VCR: vincristine
ADR: adriamycin
SDNS: steroid-dependent nephrotic syndrome
FAK: focal adhesion kinase
MCNS: minimal change nephrotic syndrome
FSGS: focal segmental glomerulosclerosis
PFA: paraformaldehyde
RT: room temperature
